# Evolutionary interaction between W/Y chromosome and transposable elements

**DOI:** 10.1007/s10709-016-9895-0

**Published:** 2016-03-21

**Authors:** Ewa B. Śliwińska, Rafał Martyka, Piotr Tryjanowski

**Affiliations:** Institute of Zoology, Poznań University of Life Sciences, Wojska Polskiego 71C, 60-625 Poznań, Poland; Institute of Nature Conservation, Polish Academy of Sciences, Mickiewicza 33, 31-120 Kraków, Poland

**Keywords:** Y chromosome, Non-recombining chromosome, Genome defense, PIWI proteins, piRNA, Transposable elements

## Abstract

The W/Y chromosome is unique among chromosomes as it does not recombine in its mature form. The main side effect of cessation of recombination is evolutionary instability and degeneration of the W/Y chromosome, or frequent W/Y chromosome turnovers. Another important feature of W/Y chromosome degeneration is transposable element (TEs) accumulation. Transposon accumulation has been confirmed for all W/Y chromosomes that have been sequenced so far. Models of W/Y chromosome instability include the assemblage of deleterious mutations in protein coding genes, but do not include the influence of transposable elements that are accumulated gradually in the non-recombining genome. The multiple roles of genomic TEs, and the interactions between retrotransposons and genome defense proteins are currently being studied intensively. Small RNAs originating from retrotransposon transcripts appear to be, in some cases, the only mediators of W/Y chromosome function. Based on the review of the most recent publications, we present knowledge on W/Y evolution in relation to retrotransposable element accumulation.

## Introduction

The W and Y chromosomes differ from the other chromosomes, mainly as they do not have homologous partners and do not recombine in their mature form (Charlesworth 1996; Rice 1996). The W/Y chromosomes evolved to prevent recombination between genes involved in the primary sex-determination process, in order to avoid production of neuters (Charlesworth 1996). Thus, at the evolutionary beginning of heteromorphic sex chromosomes, the key sex-determining and sexually antagonistic genes were physically separated onto two sex chromosomes: proto-X and proto-Y (Muller 1918; Charlesworth 1996). Once genetic sex determination is located on a heteromorphic chromosomal system, selection for alleles that are advantageous in males but disadvantageous to females can lead to further genetic differentiation between the two sex chromosomes at other loci. In consequence, a suppression of recombination may take place over most or all of the proto-Y chromosome length (Rice 1996). Such suppression of recombination allows the preservation of beneficial epistatic interactions between sexually antagonistic and sex-determination genes (Charlesworth et al. 2005).

However, the main side effect of the cessation of recombination is evolutionary instability and degeneration of the W/Y chromosome (Charlesworth and Charlesworth 2000; Charlesworth et al. 2005; Bachtrog et al. 2008; Malone and Oliver 2008; Miura et al. 2012; Sun and Heitman 2012; Bachtrog 2013) or frequent W/Y chromosome turnovers (Traut 2010; Dübendorfer et al. 2002; Blaser et al. 2013). W/Y chromosome evolutionary degeneration seems to consist of two different processes: functional and physical degradation. Physical degradation of the W/Y chromosome may become apparent in cases in which the W/Y chromosome and the Z/X chromosome evolve from a homomorphic pair of autosomes. This means that the W/Y chromosome is initially the same size as the Z/X chromosome and then often grows shorter or longer over the course of evolutionary time (Charlesworth et al. 2005). The W/Y functional degradation process involves the accumulation of deleterious mutations and a decrease in the expression of genes (Charlesworth and Charlesworth 2000), and models describing this process are still being discussed (Charlesworth and Charlesworth 2000; Bachtrog 2008, 2013; Bachtrog et al. 2008; Blaser et al. 2013). Further observations of W/Y chromosome degeneration show that there is an increase in repetitive non-coding sequences and transposon load (Charlesworth et al. 1994; Charlesworth and Charlesworth 2000; Erlandsson et al. 2000; Peichel et al. 2004; Bachtrog et al. 2008; Carvalho et al. 2009; Bachtrog 2013). The process of degeneration and rearrangement of the W/Y chromosome is an important problem that should be recognized to understand W/Y chromosome evolution (Charlesworth and Charlesworth 2000; Bachtrog 2008; Carvalho et al. 2009; Blaser et al. 2013).

However, a key and still neglected feature of W/Y chromosome degeneration is transposable element accumulation. It has been predicted that increasing transposable element load in the W/Y genome should occur as a result of recombination deficiency (Bachtrog et al. 2008; Blumenstiel 2011; Bachtrog 2013). TEs may play a significant role in the process of chromosome differentiation. This results from the fact that they allow W/Y chromosomes to achieve a state of beneficial non-homology in a short time (Charlesworth and Charlesworth 2000; Bachtrog et al. 2008). Thus, TEs may widen the non-homological regions through insertions, and enable large inversions (McDonald 1993; Charlesworth et al. 2005; Hua-Van et al. 2005; Bachtrog et al. 2008). Transposon accumulation has been confirmed for all W/Y chromosomes that have been sequenced so far (Bachtrog 2013).

The aim of this paper is to highlight the need for the involvement of specific features of TEs to the models of W/Y chromosome evolution. We discuss the current concepts of W/Y chromosome degeneration within the context of two issues concerning transposable element evolution in genomes: transposable element accumulation, and the characteristics of genome defense against transposable element invasion. Regarding these issues, we attempt to delineate a general picture of the evolutionary process (i.e., evolutionary cycle) that each W/Y chromosome goes through. As TEs are a substantial part of the W/Y chromosome (Bachtrog et al. 2008) such a discussion may be particularly important in the context of W/Y chromosome degeneration.

## Models of Y chromosome degeneration

Models of population processes proposed for explaining Y chromosome degeneration generally assume that fixation probabilities for a deleterious mutation are limited to small population sizes, or very high variances in male reproductive success (Charlesworth and Charlesworth 2000). Different cases for these assumptions were discussed by Charlesworth and Charlesworth (2000) and reviewed by Bachtrog (2013). In the work of Bachtrog et al. (2008) and Bachtrog (2013), the discussion was expanded to include the temporal dynamics for Y chromosome degeneration in terms of non-linear gene decay. The exact rate of degeneration depends on several species-specific factors, such as effective population size, number of genes present on the neo-Y chromosome, and generation time (reviewed in Bachtrog 2013). All these models assume accumulation of deleterious mutations in coding genes. Blaser et al. (2013) also proposed a model of deleterious mutation accumulation to explain frequent W/Y chromosome turnover as a result of Muller’s ratchet [random loss of haplotypic classes that display the fewest mutations (Charlesworth and Charlesworth 2000)]. The authors showed, using a simulation model, that the rapid W/Y chromosome turnover observed in most cold-blooded vertebrates might be explained by gradually increasing deleterious mutation load on the W/Y chromosome. The deleterious effect of a mutation located on the W/Y chromosome exceeds the benefits which stem from sexually antagonistic genes established in the non-recombining region and affects some fitness components of heterogametic sex. Thus W/Y chromosome replacement should be selectively favored. Blaser et al. (2013) also proposed that some W/Y chromosomal rearrangements, including the creation of a neo-Y chromosome from the autosome, may be fixed in populations because chromosomal rearrangements allow genomic blocks with a deleterious load to be discarded. However, Blaser et al. (2013), in their reasoning, took into account deleterious mutations in functional genes lacking dosage compensation. These conclusions limit the applicability of the model to species with no dosage compensation of functional genes located on the Z/X chromosome (Blaser et al. 2013). Below, we present a synopsis of the newest molecular findings that may indicate that the Blaser et al. (2013) concept of deleterious mutation accumulation for the explanation of frequent W/Y chromosome turnover may be applied to many W/Y chromosome evolutionary instability cases, regardless of the presence of dosage compensation for functional genes.

## The deleterious effect of jumping transposon load in genomes

**Fig. 1:**
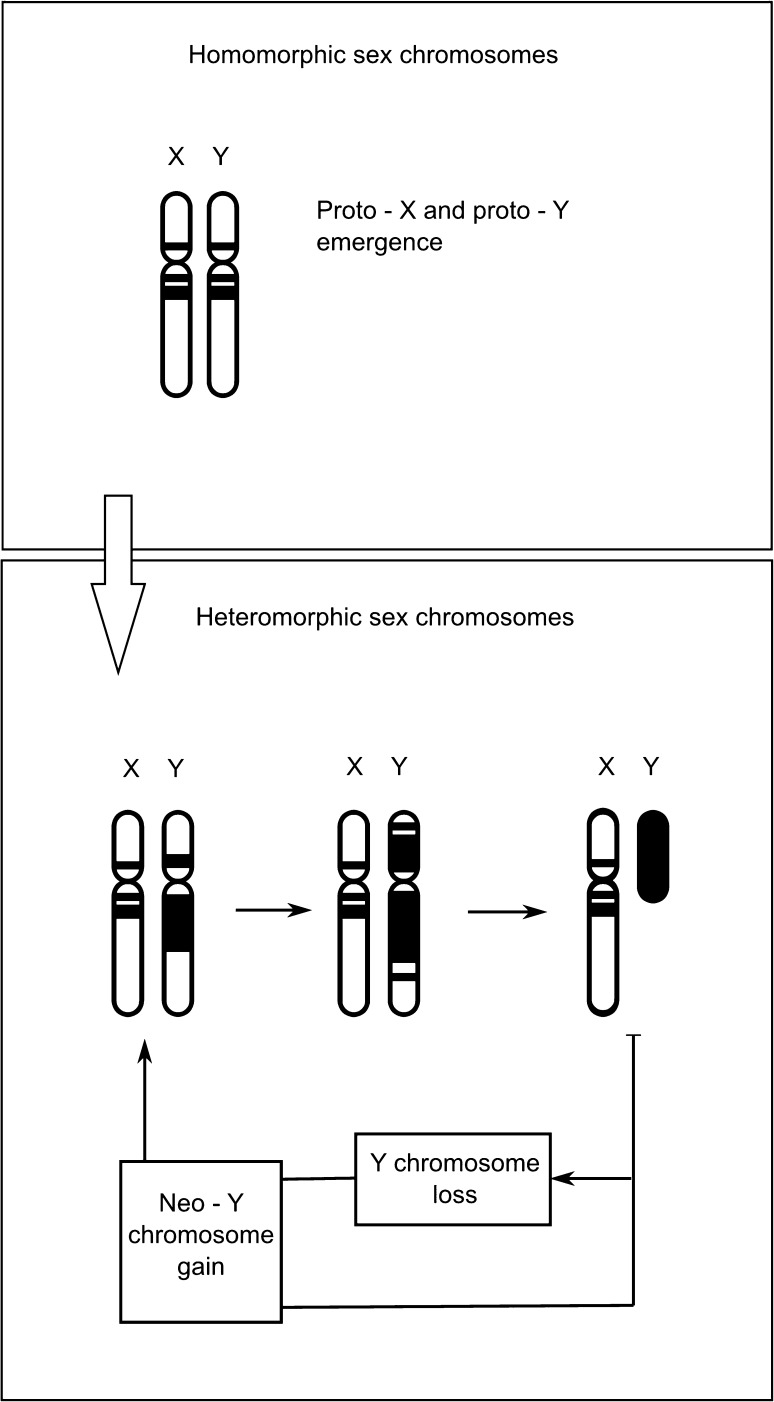
Accumulation of TEs on W/Y chromosomes. The non-recombining W/Y chromosome is colonized by transposable elements (TEs; *black bands* and *areas*) during its evolutionary degeneration. The figure presents possible stages of colonization. The *upper box* represents the homomorphic stage of sex chromosome evolution. At this stage both sex chromosomes have an identical load of TEs, similar to autosomal chromosomes. Because of ongoing recombination between proto-X and proto-Y the fixation of active and detrimental TE insertions is limited (Blumenstiel 2011; Hua-Van et al. 2011). The *bottom box* shows the heteromorphic stage of sex chromosome evolution as the Y chromosome became mature and subsequently degenerate. When areas of recombination cessation start to spread over the Y chromosome, TE insertions begin to be more successful (first cycle step in the *bottom box*). New TE insertions on the Y chromosome may fix in the population due to several processes: the hitchhiking effect of favorable mutations, Muller’s ratchet enforcing successful transposition, and the mode of interaction with silencing proteins (see text). Then, on the Y chromosome, and generally in the cell, the load of active TE insertions producing detrimental transcripts grows. Due to the failure of genomic defense against TE transcripts, the other processes leading to active TE removal start to act. In this scenario we may observe chromosome contraction, Y chromosome loss, and chromosome rearrangements, followed by neo-Y chromosome emergence (third step and the rest of the cycle in the *bottom box*). All observed rearrangements lead to the loss of blocks of active TEs

Models explaining W/Y chromosome degeneration have neglected the important process of the accumulation of functional (i.e., transposition-competent) TEs. In Fig. 1, we show a theoretical view of transposable element accumulation on the Y chromosome, a cell reaction that may occur, and a possible scenario of W/Y evolution in response to transposable element accumulation. In this process, functional TEs with the ability to transpose might present a detrimental effect on fitness, since only TEs in that state may produce copies that are able to insert and evoke a host genomic mutation (Hua-Van et al. 2005; 2011). Functional TEs also pose a constant challenge to genome defense due to transcript production (Blumenstiel 2011). It could be expected that silenced transposable element transcript load is significant and challenging to genome defense as it has been shown in the mammalian transcriptome that 6–30 % of cap-selected transcripts were initiated in repetitive elements, and 2–16 % in retrotransposons (Faulkner et al. 2009).

Retrotransposons appear to have greater significance than other transposons in their potential for deleterious effects on host fitness (Faulkner et al. 2009). Retrotransposons undergo a unique DNA synthesis process called reverse transcription. In this process, single-stranded RNA is converted into double-stranded DNA. Double-stranded DNA is then integrated into host genomic DNA (reviewed in Wilhelm and Wilhelm 2001). In their functional state, retrotransposons produce transcripts able to populate the genome, and their insertion is irreversible (Hua-Van et al. 2005). Two families of autonomously replicating retrotransposons (LINE-1 Long Interspersed Nuclear Elements, and HERVs Human Endogenous Retroviruses) together account for about 28 % of the human genome (Hua-Van et al. 2005). Around 40 % of the mouse genome consists of autonomous and non-autonomous retrotransposable elements (Mourier 2011). Retrotransposons may constitute ~90 % of all TEs accumulated on the Y chromosome (Bachtrog et al. 2008).

Functional retrotransposon inserts produce transcripts that are silenced by genome defense proteins (PIWI proteins, Box 1). Deleterious mutations in the form of functional TEs may accumulate on the W/Y chromosome initially in an inactive form, under the cover of silencing proteins. A growing number of functional insertions may only be marked by the increasing energetic costs of genome defense (see below). Further components of their deleterious value result from the risk of ectopic recombinations, insertional mutagenesis, and providing enzymatic activities for other mobile DNA elements with an effect on fitness (Blumenstiel 2011; Solyom and Kazazian 2012). Estimations show that, on average, transposable element insertion decreases the fitness of an individual by 0.4 % (Pasyukova et al. 2004).

**Box 1:** Genome defense (host-mediated transposable element regulation)

*Types of genome defense*
The genome defense system is, besides natural selection, the main force limiting transposable element spread in the genome (Galagan and Selker 2004; Blumenstiel 2011). In the living world, we can distinguish two main types of genome defense systems that have been described so far. The first is the RNA-mediated silencing system that is widespread in eukaryotes (Blumenstiel 2011). The second is the repeat-induced point mutation process (RIP), which is characteristic of some fungal species (Galagan and Selker 2004)
RNA-mediated silencing involves the production of small RNAs using the transcripts of the TEs themselves. The transposable element may insert into the genome in two orientations. This feature of TEs results in the production of sense and antisense RNA transcripts that may form double-stranded aberrant RNAs. These particles are recognized by cell systems and cut into small 21–30 nt RNAs. Small RNAs join with the group of Argonaute (AGO) proteins. More detailed information about the phylogenies and function of AGO proteins in the living world can be found in Peters and Meister (2007), Seto et al. (2007), Thomson and Lin (2009), Senti and Brennecke (2010), and Siomi et al. (2011)
Generally, among small RNAs involved in genome defense systems, there are distinguishable small-interfering RNAs (siRNAs) that are characteristic for plants, and PIWI-interacting RNAs (piRNAs) that are characteristic for animal gonads. siRNAs are produced in all plant tissues from double-stranded RNA processed by the Dicer endoribonuclease. They repress TEs primarily through RNA-directed DNA methylation (Matzke et al. 2009). In contrast to siRNAs, animal piRNAs are Dicer-independent, interact with PIWI proteins (a specialized group of AGO proteins), and are produced only in germline tissues. Both types of small RNAs repress TEs through post-transcriptional gene silencing (PTGS) and transcriptional silencing (TGS) by DNA methylation and heterochromatin formation
The RIP genome defense is an extremely efficient mechanism against transposable element spreading throughout the genome of filamentous fungi (*Neurospora crassa*, Galagan and Selker (2004), review). It acts only during the sexual cycle and identifies all duplicated sequences, regardless of origin. After identification of duplicated regions greater than 400 bp, it introduces C:G to T:A mutations into both copies of duplicated sequences. RIP-mutated sequences are then often targets for DNA methylation in vegetative cells, similar to those in mammals and plants. The protein responsible for RIP activity is DNA methyltransferase-like enzyme (DMT)
*Repressor allele evolution*—*phases of transposable element invasion* (*after Blumenstiel (* 2011 *) review*)
A repressor allele is a transposable element allele: they produce small RNAs used by genome defense systems to silence transposable element transcripts from the same transposable element family. Repressor alleles can evolve during transposable element invasion and can be fixed in the host genome during the process of adaptation of the host genome to the invasion. In the most common cases, the current knowledge allows us to distinguish four phases of transposable element invasion into the genome. The initial phase involves transposable elements spreading between individuals within a population. The second phase is the multiplication of transposable element copies in the genome, within the population. During the third phase, TE repressor alleles appear in the genome that can initiate the production of small silencing RNAs. The repressor alleles are then fixed in the population and spread throughout the genome; this results in the repression of other members of the transposable element family. In the fourth phase, stabilization of transposable element copy number, and repression of transposition rate of a given transposable element family occurs. More detailed information is available in Blumenstiel (2011)

Models of transposable element dynamics in the genome are reviewed in depth in Hua-Van et al. (2011). The evolution and dynamics of TEs in the genome are shaped by a balance between transposition and selection. It is assumed that TEs are slightly deleterious and decrease host fitness: therefore, they tend to be eliminated by purging selection. At the same time, the transposition process tends to increase the copy number, as this is advantageous for TEs. To explain transposable element existence in the genome during the longer period of evolution, there is no need for equilibrium between these two opposing processes. Sudden changes disrupting this equilibrium are observed, for example, during bursts of transposition (Hua-Van et al. 2011). The other non-adaptive forces responsible for transposable element persistence in the genome are effective population size and the mode of host reproduction. Selection against TEs is less effective in small populations because genetic drift is stronger as effective population size decreases. The invasive properties of TEs includes their ability to multiply within one genome and to spread within the population (Hua-Van et al. 2011). Without sexual reproduction, transposons do not spread to the genomes of other individuals. Therefore, transposons are unable to populate genomes in populations of asexual taxa (Arkhipova and Meselson 2000).

Including W/Y chromosome and genome defense characteristics in the model of transposable element dynamics in the genome adds another dimension to understanding transposable element invasion. In our reasoning, we try to use some features of the model of Badge and Brookfield (1997) by including host factors such as genome defense and W/Y chromosome characteristics. Taking into account these host factors, we may see that some of the deleterious effect of TEs on host fitness might not be constant over time. In the case of retrotransposons, each new insertion may slightly weaken the silencing effectiveness of genome defense on all other functional transcripts, and/or increase the energetic expense of cells, as additional proteins need to be synthesized to maintain genome defense efficiency. Total genome defense costs increase with each new source of functional transposable element transcripts. After each insertion of functional TEs, the negative selection coefficient can grow for every other functional silenced element present in the genome at a given time. Energetic costs of genome defense may increase with time as the number of functional TEs producing transcripts grows. In other words, we suggest that the exhaustion of host factors (proteins of genome defense) through increasing numbers of binding sites in retrotransposon transcripts may lower host fitness, first, through the unavailability of host factors required for other processes (see below), and second, by the finite availability of host factors (Badge and Brookfield 1997). The model proposed by Badge and Brookfield (1997) is a conception assuming that host factors facilitate transposition, instead we propose existing host factors limit successful transposition. Such limiting host factors would be genome defense proteins. In turn, on the non-recombining chromosome, host factors may enforce successful transposition (i.e., bursts of transposition) by providing a suitable environment for transposable element accumulation.

Finally, our conclusion is that the concept of W/Y chromosome evolution should take into account both cumulative transposable element effects on fitness in terms of energetic costs for cell systems and functional sequence disruption by TEs. For functional TEs located on the W/Y chromosome, the net selection coefficient for deleterious mutations should grow with functional transposable element accumulation up to the point where almost all, or all of the W/Y chromosome has a detrimental effect on fitness (Blaser et al. 2013).

## Genome defense against transposable element invasion

So far, it has been documented that the genome may be protected against overpopulation of selfish elements in several ways, which we present in Box 1. The most widely occurring among species, and at the forefront of genome defense, is RNA silencing (Siomi et al. 2011). The multi-functionality of genome defense proteins and their specialization level (Siomi et al. 2011) may constitute some limitations in the defense of the genome, depending on the potency of transposable element invasion. For example, the multi-functionality of defense proteins [their participation in gene silencing in different cell processes (Siomi et al. 2011)] guarantees that the system of genome defense proteins in the cell is always ready to protect the genome. However, when transposable element invasion reaches certain level of potency, the multi-functionality of the defense proteins may present a limitation in their ability to react effectively. As they are engaged in other cell functions, effective transposable element transcript silencing may be impaired. We postulate that the general effectiveness of genome defense is not perfect as a relatively significant load of TEs and their remnants in the genome may be observed in eukaryotes (Hua-Van et al. 2005; Bachtrog et al. 2008; Matzke et al. 2009). The most efficient genome defense appears to be expressed by filamentous fungi (RIP genome defense, see Box 1), but some relicts of transposable element insertions are still present in their genomes (Galagan and Selker 2004), particularly on the non-recombining mating-type chromosome (Menkis et al. 2008).

Besides transposable element silencing, genome defense proteins are involved in other cell processes contributing to repetitive DNA methylation (Seto et al. 2007; Siomi et al. 2011). In many different organisms, PIWI proteins play important roles from the earliest stage of germline development (germline fate specification) to late stages of gametogenesis, egg activation, and fertilization (reviewed in Thomson and Lin 2009). In plants, genome defense proteins are non-specific [Dicer and Argonaute proteins (Seto et al. 2007)]. In contrast, animal proteins that silence TEs are highly specialized, such as the PIWI protein group. However, these proteins still play important roles in many different aspects of physiology. The PIWI proteins interact with piRNAs most often only in germ tissue and in particular phases of the germline cycle (Thomson and Lin 2009).

Overall, we presume that the enzymatic character of genome defense creates energetic costs that may limit genome defense preparedness during (over generations) a massive increase in transposable element transcripts in the cell. We draw attention to the process of transposable element accumulation on the W/Y chromosome as it may lead to genome defense failure in some particular evolutionary circumstances. RNA silencing genome defense is composed of proteins that interact primarily with transposable element transcripts, but also express methylation activity of functional transposable element sequences (Siomi et al. 2011). We assume that some fraction of the functional transposable element sequences located specifically on the W/Y chromosome may not be silenced by methylation as long as the genes under positive selection remain on this chromosome. This is because there is little scope for removing insertions that are closely linked to these genes as the chromosomes do not recombine. Methylated transposable element insertions in the close neighborhood of these genes may also create additional deleterious effects on fitness (Hollister and Gaut 2009). This situation presents an evolutionary window for selfish elements to produce functional transcripts and spread throughout the rest of the genome. Therefore, due to functional transposable element accumulation on the W/Y chromosome, genome defense may be more stressed from one generation to the next. The cell must expend more on genome defense proteins from one generation to the next because the previous levels of their activity were insufficient. Thus, genome defense might be costly in the context of the non-recombining chromosome.

During transposable element invasion, the simultaneous occurrence of other processes in the cell that result in increased demand for the production of PIWI proteins may also elicit additional energetic costs of genome defense. As a result of genome defense failure following transposable element transcript accumulation, the transcripts slip out of the silencing system and more frequent successful transposition should be observed. One of the most interesting consequences of genome defense system failure may be transposable element induced non-adaptive phenotypic plasticity. Developmental robustness, is a feature of an organism, it consists of the ability to produce the same phenotype despite genotypic variations and environmental influences: it is known as ‘canalization’. The recent work of Gangaraju et al. (2011) has shown the molecular mechanism involved in canalization in Drosophila. This mechanism involves the piRNA pathway and a protein complex composed of Hsp90, PIWI, and the Hsp70/Hsp90 Organizing Protein Homolog (Hop). These authors demonstrated the role of this complex in the mediation of canalization, its role in epigenetic silencing of the expression of existing genetic variants, and, most interestingly, the suppression of new genetic variation induced by transposons. Furthermore, Specchia et al. (2010) showed in Drosophila that functional alterations of Hsp90 affect the PIWI-interacting RNA silencing mechanism, and that this process led to transposon activation and the induction of morphological mutants. The conclusion from the work of Specchia et al. (2010) is that Hsp90 mutations can generate new variation by transposon-mediated ‘canonical’ mutagenesis. Before Gangaraju et al. (2011) and Specchia et al. (2010), it was known that in both flies and plants, mutations in the Hsp90-encoding gene induce a wide range of phenotypic abnormalities, which have been interpreted as an increased sensitivity of different developmental pathways to hidden genetic variability (Queitsch et al. 2002).

The above findings support our idea that transposable element transcript invasion in a cell (as a result of W/Y chromosome aging) may lead to increased expression of the PIWI protein complex, and thus entails some energetic costs for cell systems. Because the PIWI and Hsp90 heat-shock proteins also have other functions in addition to transposable element transcript silencing, in situations of environmental or internal stress the level of their expression may not be high enough to maintain genome defense at the required level (Vasil’eva et al. 2011).

Functional TEs located on the W/Y chromosome may therefore present a ticking bomb with no or little effect on fitness until genome defense is overloaded. We postulate that in some cases of environmental or internal stress, genome defense failure may be a relatively sudden process. After which a great number of functional transposable element insertions on the W/Y chromosome may produce transcripts that accumulate in a very short time and insert throughout the genome. The maladaptive mutations, hitherto silenced by the genome defense system, might be uncovered ‘all at once’, i.e., within a number of generations not large enough for the genome defense system to adapt.

After genome defense failure, the other processes of transposable element removal begin. Such processes can frequently be observed as different forms of W/Y degeneration. The mechanisms of deleterious load removal may include W/Y chromosome heterochromatization, contraction, and loss. The fusion of the old Y chromosome with the autosomal genome or X chromosome may also be a way to remove huge blocks of functional TEs from the genome. The neo-Y chromosome may be observed as a final side-effect of that process (Blaser et al. 2013). Considering the common and wide occurrence of W/Y chromosome rearrangements, and their fixation among a substantial number of investigated species, W/Y chromosome contraction and loss may be an adaptive process to regain fitness (Blaser et al. 2013).

## Conception of the evolutionary cycle of the W/Y chromosome

The beginning of transposable element accumulation on the W/Y chromosome (Fig. 1) may start from several insertions in the close neighborhood of crucial heterogametic sex-linked genes. This position guarantees that functional TEs can escape the removal from the population due to stochastic population processes, methylation/heterochromatization (Hollister and Gaut 2009), or Y chromosome contraction. The mechanism is called the hitchhiking effect of favorable mutations (Charlesworth and Charlesworth 2000). The linkage of a transposon insertion with a locus under strong positive selection would generate the conditions required for this mechanism. Additionally, the restriction of recombination on the W/Y chromosome may create hundreds of linked genes. Therefore purifying selection, acting against deleterious mutations, may be greatly reduced (Bachtrog 2013).

In the presence of an efficiently working genome defense, functional TEs may cause a weak deleterious effect on general fitness: therefore, functional insertions would be easily fixed in the population. These functional insertions in the close neighborhood of heterogametic genes might be a germ for the overpopulation of W/Y chromosome. The growth of TEs blocks on the W/Y chromosome at the beginning of the overpopulation process should be relatively slow (the rate of transposition should be similar to autosomal chromosomes) and limited by the silencing of transposable element transcripts. Then, the widening of non-recombining regions with non-coding host sequences, could allow an increase in the number of successful transpositions. TEs may insert within other transposable element sequences, within both functional and already degenerated elements (so-called nesting (Hua-Van et al. 2005)), and in this way increase the total number of actively transcribing and transposing copies [e.g., transposable element composition in the *Bombyx mori* W chromosome (Abe et al. 2005)].

Furthermore, TEs linked to the heterogametic sex-determining genes should be the most active and successful because they possess the ability to produce functional transcripts over long evolutionary time scales (reflecting the view of the genome as an ecosystem with TEs as individual members of a species, reviewed in Hua-Van et al. 2011). Such processes may result from slower pseudogenisation and heterochromatization of functional TEs located in the neighborhood of functionally important genes in the condition of no-recombination (Mourier and Willerslev 2010). Further generations of such TEs may overpopulate the W/Y chromosome and then spread throughout the genome. In turn, this would contribute to the observed effect of ‘concerted evolution’ of repetitive elements in genomes (Elder and Turner 1995), because only a few elements placed in close neighborhood of functional genes may spread their copies in the whole genome.

The subsequent cycles of W/Y chromosome degeneration and rejuvenation (Fig. 1) may differ in their length of evolutionary time. Cycles should begin spontaneously at any time or start after a few to a few hundred million years as suggested by the estimated age of W/Y chromosomes across species (Box 2). We propose that during W/Y chromosome degeneration, the appearance of a significant number of transposable element transcripts in a cell might be more or less sudden. In species/populations with a fixed W/Y chromosome loss or with an observed frequent turnover, the period of TE transcript increase may be too short to induce trans-generational and constant preparedness of the genome defense system for transposable element invasion. When genome defense is inefficient, W/Y chromosome degradation may be quicker and the cycle of chromosome rejuvenation shorter.

**Box 2:** W and Y chromosome age and degeneration status

Taxa	Sex-determination system	Degeneration of W/Y (depictively)	W/Y chromosome age^a^	References
Filamentous fungi *Neurospora tetrasperma*	No sex chromosomes Non-recombining mating-type chromosome. Pseudohomothallism	75 % of mating-type chromosomes do not recombine Observed similar process of degeneration as in W/Y chromosome (including accumulation of transposable elements and gene pseudogenisation)	3.5–5.8 MY^b,c,d^	Thomson and Lin (2009)
Plants White campion *Silene latifolia*	Heteromorphic homologic XY, GSD	20 % of genes are lost on the Y chromosome. Accumulation of transposable elements	Oldest stratum 10 MY^d^	Bergero and Charlesworth (2011) and Chibalina and Filatov (2011)
Lepidoptera *Bombyx mori*	Heteromorphic WZ, GSD	No protein-coding genes on W chromosome >579 genes on previously homologous Z chromosome	90–100 MY^b,c^	Fujii et al. (2010), Hara et al. (2012), Sahara et al. (2012)
Diptera Phorid fly *Megaselia scalaris*	Homomorphic XY, GSD	Very early molecular signs of chromosome differentiation Rapid Y chromosome turnover within the species	Variable	Traut (2010)
Diptera Housefly *Musca domestica*	Homo- or heteromorphic XY, GSD	Early molecular signs of chromosome differentiation Y chromosome turnover within the species	Variable	Blaser et al. (2013)
Diptera *Drosophila albomicans*	Heteromorphic non-homologic XY, GSD	Neo-Y chromosome with no obvious signs of degeneration. ~4800 genes are still functional	0.12 MY^d^	Bachtrog (2006) and Zhou et al. (2012)
Diptera *Drosophila miranda*	Heteromorphic non-homologic XY, GSD	Neo-Y chromosome ~50 % of transposable elements, 209 putative genes left (~10 % of initial gene number) Transposable elements on 1 % of the neo-X chromosome	1.2 MY^d^	Bachtrog et al. (2008) and Steinemann and Steinemann (2005)
Diptera *Drosophila pseudoobscura*	Heteromorphic non-homologic XY, GSD	No protein-coding genes on Y chromosome (initially ~3000)	15 MY^d^	Carvalho and Clark (2005)
Fish Family Adrianichthyidae Medaka fish *Oryzias latipes*	Homomorphic homologic XY, GSD and TSD	Y chromosome degenerated only in 258 kb long sequence. The rest of the chromosome is homologous to X Mechanism of recombination of the male-specific region is preventing the spread of the non-recombining region over the Y chromosome	10 MY^d^	Matsuda (2005), Kondo et al. (2006), Herpin and Schartl (2009)
Fish Family Gasterosteidae Stickleback fish, *Gasterosteus aculeatus*	Heteromorphic homologic XY, GSD, environmental SD	64 % of homology among X and Y specific contigs Multiple duplications and insertions, insertions of transposons and other repeated sequences on Y chromosome	10 MY^d^	Carvalho et al. (2009)
Amphibia Family Hylidae *Hyla arborea*, *H. intermedia*, and *H. molleri*	Homomorphic homologic XY or WZ, TSD	Degeneration of Y chromosome is prevented by rare recombination with X in phenotypic females Y or W chromosome are evolutionary stable (‘fountain-of-youth’ hypothesis)	5.4–7.1 MY^d^	Stöck et al. (2011)
Amphibia Family Bufonidae *Bufo siculus*, *B. balearicus*, *B. turanensis* and *B. shaartusiensis*	Homomorphic homologic XY, TSD	Y chromosome degeneration probably prevented by rare male recombination of X and Y. Y chromosome is evolutionary stable (‘fountain-of-youth’ hypothesis)	3.3 MY^d^	Stöck et al. (2013)
Reptiles Family Viperidae Pygmy rattlesnake *Sistrurus miliarius*	Heteromorphic homologic WZ, GSD	61 W-linked genes and 712 Z-linked genes. Accumulation of repetitive elements on the W chromosome	≥50 MY^c,d^	Vicoso et al. (2013)
Reptiles Family Colubridae Garter snake *Thamnophis elegans*	Heteromorphic homologic WZ, GSD	29 W-linked genes and 723 Z-linked genes. Accumulation of repetitive elements on the W chromosome	≥50 MY^c,d^	Vicoso et al. (2013)
Birds	Heteromorphic homologic WZ, GSD or environmental SD	Different lineages represent different stages of W degradation Number of genes on the W chromosome is tens to 100, while on the Z chromosome ~1000 The W chromosome is a degenerate relict of Z and is the same among species. No W turnovers observed	120 MY^c,d^	Wright et al. (2012), Graves (2014), Wright et al. (2014)
Mammals	Heteromorphic, homologic XY, GSD	Y chromosome is more degraded than avian W chromosome On the Y chromosome, a few dozen genes are observed while on X ~1000 genes The Y chromosome is a degenerate relict of X. Rare cases of Y turnover or absence are observed among taxa	>200 MY^c,d^	Graves (2006), Veyrunes et al. (2008), Bachtrog (2013), Bellott et al. (2014)
*Homo sapiens*	Heteromorphic, homologic XY, GSD	On the Y chromosome, 86 genes have been observed, while on X 1098 genes No difference in TE percentage (44 vs. 54 %) on X- and Y-linked zinc finger genes	Five evolutionary strata on the Y chromosome^d^	Skaletsky et al. (2003), Peichel et al. (2004), Ross et al. (2005), Goto et al. (2009)

Listed examples of W and Y chromosome estimated age, from different taxa are shown below. The sex determination system and available information on chromosome degeneration are given. The listing includes the relevant literature

*SD* sex determination, *GSD* genetic sex determination, *TSD* temperature sex determination, *MY* million years

^a^Different ways to obtain W/Y chromosome age since recombination stopped

^b^Degree of heteromorphism

^c^Age of the group of species

^d^X–Y or neo-Y—autosome divergence study [after Charlesworth (2012)]

We suggest that the W/Y chromosome probably becomes a substantial source of functional transposable element transcripts in the cell, and therefore, may present a threat to the stability of the whole genome. In fact, observations of rapid transposable element accumulation on the W/Y chromosome (Charlesworth et al. 2005) and the few available comparisons of transposable element load on autosomal, X, and Y chromosomes (Pimpinelli et al. 1995; Abe et al. 2005; Bachtrog et al. 2008; Matzke et al. 2009; Piergentili 2010) have shown that the W/Y chromosome may be a genomic source of functional TEs. However, the cumulative load of functional TEs from different transposable element groups has not yet been investigated in detail, on the W/Y or other chromosomes (Hua-Van et al. 2005; Piergentili 2010). This is probably due to difficulties in W/Y chromosome sequencing (Carvalho et al. 2009; Bachtrog 2013), limitations of the available transposable element searching software (Hua-Van et al. 2005; Bachtrog et al. 2008), and difficulties in the assignation of functional transposable element transcripts to particular loci (Mourier and Willerslev 2010). Due to these limitations, we expect that the current transposition rate of particular transposable element families (Nuzhdin and Mackay 1995; Charlesworth and Charlesworth 2000) may be underestimated and/or artificially standardized over evolutionary time. A more useful point of view may take into consideration the fluctuation of the transposition rate (Blumenstiel 2011).

One of the interesting exceptions from the proposed evolutionary cycle of non-recombining chromosomes, is Y chromosome evolution in *Oncorhynchus*, a genus of fish within the family Salmonidae. *Oncorhynchus* fish possess an XX–XY sex determination system with some populations having morphologically distinct Y chromosomes (Thorgaard 1978). Among *Oncorhynchus* species there are six independent sex-chromosome pairs aged 6–8 MYA (Lubieniecki et al. 2015). The variety of Y chromosomes most probably results from the sex determining region’s (SDR) ability to move throughout the genome (Faber-Hammond et al. 2015). The mechanism of SDR movement is proposed to involve transposons which flank the sex determination region. There are two main transposon candidates responsible for the novel SDR insertions: TC1-like transposase and RNA-directed DNA polymerase from mobile jockey-like elements (non-LTR retrotransposon, Faber-Hammond et al. 2015). TC1-like elements transpose via double strand DNA breaks. It is possible that instead of targeting an element for transposition, transposase enzymes move the large SDR flanking region throughout the genome. In the case of jockey-like elements, a possible mechanism of SDR movement may be through RNA templates. RNA-directed DNA polymerase reverse transcribes, and inserts the SDR cassette at a new genomic locus. In this case jockey-like elements could serve as a terminator sequence for transposition, a site of insertion of the SDR sequence into a genomic target, and a site of initiation for reverse transcription of the SDR. Although all proposed mechanisms are speculative, the movement of the male sex determination region among autosomal chromosomes may provide a way out for the problem of Y chromosome degradation and deleterious transposon accumulation. The young age and the number of new Y chromosomes in *Oncorhynchus* taxa may indicate the presence of an evolutionary mechanism which allows for quick rejuvenation of degrading Y chromosomes with gradually enlarging non-recombining regions.

## Transposons as alternative mediators of W/Y chromosome function

The role of the W/Y chromosome as a regulator of many phenotypic traits has been documented recently (Piergentili 2010; Hara et al. 2012; Branco et al. 2013; Sackton and Hartl 2013; Lokody 2014). The discovery of the regulation mechanisms of many autosomally encoded genes in *Drosophila melanogaster* and *D. simulans* has shown that the Y chromosome is part of a network of genetic and biochemical interactions (Piergentili 2010; Branco et al. 2013). Furthermore, the discovery of sex determination in *Bombyx mori* demonstrates the regulatory function of the W chromosome (Kawaoka et al. 2011; Hara et al. 2012). The mechanism of regulation does not include protein-coding genes (Piergentili 2010; Kawaoka et al. 2011; Hara et al. 2012; Branco et al. 2013). Instead, repetitive sequences (i.e., TEs and satellite DNA) are involved in physical and biochemical interactions with thousands of autosomal protein-coding genes (Piergentili 2010; Kawaoka et al. 2011; Hara et al. 2012; Sackton and Hartl 2013). Transposons have also been shown to play a role in heterochromatization of the Y chromosome and dosage compensation mechanisms in *Drosophila* ssp. (Ellison and Bachtrog 2013; Zhou et al. 2013). These studies concentrated on model species, but a similar composition of the W/Y chromosome is characteristic of many Eukaryota (Piergentili 2010; Branco et al. 2013; Sackton and Hartl 2013). Repetitive elements (TEs and satellite DNA) are components of all sequenced non-recombining chromosomes. Therefore, it may be expected that the W/Y chromosome plays a substantial role in autosomal gene regulation in many other species (Piergentili 2010).

In light of recent knowledge, the role of TEs in sex chromosome evolution goes beyond the evolutionary fight against them. The W/Y chromosome degenerates to the point where there are scarce or no canonical (protein-coding) genetic effectors for its evolutionary role (Box 2): thereby, it becomes the source of potentially deleterious TE transcripts. These two facts might indicate the involvement of transposable element transcripts in the regulation of cell processes concerning heterogametic sex. Using transposable element transcripts in the synthesis of small regulatory RNAs while eliminating the potential threat to genome stability (by using genome defense proteins) would give an unexpected advantage for the W/Y chromosome’s evolutionary role. It enables a great evolutionary flexibility in such a retrotransposon-based regulatory system as rearrangements or mutations occurring on degenerating non-recombining chromosomes (including transposon-induced rearrangements and mutations) have a small or no effect on its function and ultimately the fitness of the host. Quantitative (dose-dependent) autosomal gene regulation induced by the Y chromosome in *D. melanogaster* and the W chromosomal sex-determination system of *B. mori* (Piergentili 2010; Kawaoka et al. 2011; Hara et al. 2012) present examples of such a use of transposable element transcripts which emerge from the W/Y chromosome.

## Conclusions

By reviewing the recent discoveries in the areas of non-recombining chromosome evolution, dynamics and evolution of TEs, as well as the genome defense system, we have attempted to show the interactions between these three molecular fields. In particular, we have highlighted the potential relationship between transposable element dynamics and non-recombining chromosome instability mediated by the genome defense system. TEs appear to influence W/Y chromosome fate through the accumulation of functional selfish insertions. In turn, non-recombining chromosomes may influence transposable element dynamics by allowing them to produce functional selfish copies that populate the rest of the genome. We postulate that the evolution of non-recombining chromosomes should always take into account the potential impact of TEs on fitness. Similarly, the models of transposable element dynamics and evolution should include the presence of the non-recombining genome in sexual organisms. We believe that this may allow us to fully understand W/Y chromosome evolution and function.
